# Editorial: Global dissemination and evolution of epidemic multidrug-resistant Gram-negative bacterial pathogens: surveillance, diagnosis and treatment, volume III

**DOI:** 10.3389/fmicb.2025.1583112

**Published:** 2025-03-12

**Authors:** João Pedro Rueda Furlan, Raffaele Zarrilli, Ziad Daoud, Fang He, Zhi Ruan

**Affiliations:** ^1^Paulista School of Medicine, Federal University of São Paulo, São Paulo, Brazil; ^2^Department of Public Health, School of Medicine and Surgery, University of Naples Federico II, Naples, Italy; ^3^College of Medicine, Central Michigan University, Mount Pleasant, MI, United States; ^4^Department of Clinical Microbiology and Infection Prevention, Michigan Health Clinics, Saginaw, MI, United States; ^5^Laboratory Medicine Center, Department of Clinical Laboratory, Zhejiang Provincial People's Hospital (Affiliated People's Hospital), Hangzhou Medical College, Hangzhou, Zhejiang, China; ^6^Department of Clinical Laboratory, Sir Run Run Shaw Hospital, Zhejiang University School of Medicine, Hangzhou, China

**Keywords:** bacterial infections, multidrug resistance, whole-genome sequencing, genomic epidemiology, surveillance

The global rise of multidrug-resistant (MDR) Gram-negative bacteria poses a significant public health threat. Studies have reported the widespread presence of MDR pathogens across different environments, including clinical settings, food production, and waters. Key findings highlight the role of horizontal gene transfer (HGT), mobile genetic elements (MGEs), and plasmids in antimicrobial resistance (AMR) dissemination. The emergence of strains resistant to third-generation cephalosporins, carbapenems, and polymyxins further underscores the need for continuous surveillance. Understanding AMR mechanisms, including enzymatic degradation by β-lactamases, and risk factors is crucial for effective prevention and control strategies. This Research Topic includes original research, review, and systematic review focused on the global dissemination and evolution of AMR in Gram-Negative bacterial pathogens.

## The prevalence of MDR Gram-negative bacteria

The spread of MDR bacterial pathogens is a growing global health concern. In China, Zheng L. et al. reported MDR *Salmonella* Enteritidis strains from retail meat and environmental samples from 2014 to 2019. These strains presented distinct resistance patterns, with a prevalence of MDR of more than 70%. Interestingly, a fluctuating trend likely influenced by tetracycline withdrawal management from 2015 to 2019 was observed. Genomic analysis revealed acquired and mutational mechanisms related to multidrug resistance. Furthermore, an IncX1 plasmid was detected as harboring genes related to resistance to ampicillin (*bla*_TEM_), sulfisoxazole (*sul2*), and streptomycin [*aph(6)-Id* and *aph(3*″*)-Ib*]. The antimicrobial-resistant *Salmonella* strains were primarily divided into two clusters, underscoring the potential cross-contamination within the retail chain.

Opportunist MDR bacterial pathogens are part of the environmental microbiome. Chen et al. identified MDR *Aeromonas* species in coastal waters. Strains of *Aeromonas veronii* and *Aeromonas caviae* were resistant to eleven antimicrobial agents but remained susceptible to ceftazidime. Several antimicrobial resistance genes (ARGs) and MGEs were detected by genomic analysis. Genomic islands were identified as harboring ARGs, which were related to class 1 integron and transposon. Chromosome-borne *mcr-3.16* and *mcr-3.3* (colistin resistance) were identified in *A. veronii* and *A. caviae*, respectively. These genes were flanked by insertion sequences. Furthermore, tandem regions and incomplete *mcr-3*-like genes were also observed. *In vivo* experiments classified *A. veronii* and *A. caviae* as moderately pathogenic and non-pathogenic, respectively.

In hospitals from Southern Ethiopia, Odoko et al. examined the prevalence of species of *Enterobacterales* confer resistant to third-generation cephalosporins and carbapenems by the phenotypic detection of extended-spectrum β-lactamase (ESBL) and carbapenemases. The bacterial strains exhibited multidrug resistance, with *Escherichia coli* and *Klebsiella pneumoniae* were the most frequent. Accordantly, *Enterobacteriaceae* strains producing ESBL (66.6%, 46/69) and carbapenemases (21.7%, 15/69). Among patients suspected of surgical site, multidrug resistance rate of *Enterobacteriaceae* strains was 41% (25/61). By using multivariable analysis, it was identified that the length of hospital stay was a statistically significant factor associated with surgical site infection by ESBL-positive *Enterobacteriaceae* strains. These findings highlight the importance of ongoing phenotypic and genomic surveillance to track and understand MDR bacteria.

## ESBL-producing *Enterobacteriaceae*: from a One Health perspective

Zhang et al. discussed the dissemination of genes encoding ESBLs in species from the *Enterobacteriaceae* family. Overall, ESBL-producing strains have been globally reported at the human-animal-environment interface, with *bla*_CTX − M−15_ gene being the most common ESBL-encoding gene among all reservoirs. The environment was identified as a hotspot of cephalosporin-resistant and ESBL-producing bacteria. A great diversity of plasmid families has been identified as carrying these genes, highlighting IncF, IncI1, IncK, and IncN types. Among the insertion sequences and transposons related to ESBL-encoding genes, IS*Ecp1*, IS*26*, IS*903*, IS*1380*, and Tn*3*, play a key role in the capture and mobilization of them. In this context, the need for continuous monitoring of ESBL-producing bacteria is considered desirable.

In the food production chain, ESBL-positive strains have been constantly reported. In China, Bello et al. analyzed multidrug-resistant *E. coli* recovered from dairy cows. More than 20% of *E. coli* strains were multidrug-resistant. Genomic analysis revealed several ARGs, including *bla*_CTX − M−55_, *mph(A)*, and *qnrS1*. Furthermore, MDR *E. coli* strains presented virulence factors associated with type III secretion system and adhesion. Phylogenetic analysis showed distinct evolutionary ST3579 and ST1121 lineages, suggesting that they originated from different ancestral populations. Besides, a pangenome analysis of diverse *E. coli* clones showed significant genetic diversity, with unique strain-specific genes found. MGEs were also identified, highlighting their role in bacterial diversity and adaptation.

Accordingly, Liu Y. et al. characterized ESBL-producing *Salmonella* strains from swine and broilers. *S. Typhimurium* and *S. Enteritidis* were the most prevalent serotypes in swine and broilers, respectively. The emergence of AMR was highlighted by the high percentage of multidrug resistance, which showed a downward trend over the period studied. Among the ESBL-positive *Salmonella* strains from swine, *bla*_CTX − M−14_ and *bla*_CTX − M−65_ were dominant and were harbored mainly by ST34 and ST17 clones. Poultry-derived *Salmonella* strains mainly harbored *bla*_CTX − M−55_ and *bla*_CTX − M−65_, which belonged to ST17 and ST198 clones. These findings emphasize the importance of monitoring MDR bacteria in the food production chain. Therefore, it is important to provide support for the effective prevention and control of antimicrobial-resistant strains.

## Multidrug and carbapenem resistance in *Enterobacteriaceae*: an ongoing threat to public health

The spread of carbapenem resistance in clinical strains of *Enterobacteriaceae* is largely driven by HGT, yet in-host transferability remains underexplored. The study conducted by Ji et al. analyzed strains from gut specimens of five individuals, each hosting two different species, including *E. coli, K. pneumoniae, Klebsiella aerogenes, Enterobacter cloacae*, or *Citrobacter koseri*. All strains carried a plasmid-borne carbapenemase gene (i.e., *bla*_KPC − 2_, *bla*_NDM − 1_, or *bla*_NDM − 5_). Accordingly, five plasmids were successfully transferred to recipient species. The genetic contexts of carbapenemase genes were highly similar between the two carbapenem-resistant *Enterobacteriaceae* strains from each individual, highlighting the potential for interspecies plasmid transmission within the human gut. These findings emphasize CRE colonization as a major risk factor for the dissemination of carbapenemase-encoding genes and underscore the importance of intestinal carbapenem-resistant strains screening and colonization prevention strategies.

In China, Li et al. reported a prospective study covering three periods from 2022 to 2024 describing characteristics (epidemiology, cross-transmission, interventions, and outcomes) of infections caused by carbapenem-resistant *K. pneumoniae* (CRKp) in the hematological malignancies (HM) department of a tertiary teaching hospital. Among 217 patients infected by *K. pneumoniae* strains, 38 had carbapenem-resistant *K. pneumoniae*, with ST11 harboring *bla*_KPC − 2_ gene as the predominant clone. A total of 23 HM-acquired CRKp infections and five hospital cross-transmission events were identified, with four linked to the dissemination of ST11 lineage. A single outbreak was identified at the end of Period 1 involving four healthcare-associated infections. The rate of CRKp strains declined in Period 2 when compared to Period 3. The 28-day mortality rate among CRKp-infected patients was 38.7%. The overall prevalence of CRKp in the HM department remained relatively low, and interventions such as single-room isolation, enhanced disinfection, and skin decolonization played a crucial role in controlling the spread of CRKp infections.

Idrees et al. systematically reviewed ARGs in *K. pneumoniae* exhibiting multidrug resistance in Gulf Health Council (GHC) countries. Saudi Arabia contributed the largest number of studies, followed by Kuwait, the United Arab Emirates (UAE), Oman, Qatar, and Bahrain. Considerable variability was observed in detection methods, target genes, and AMR mechanisms. Most studies focused on clinical samples, with only one environmental study in the UAE and one community-based study in Kuwait. AMR patterns varied across countries and over time, with widespread detection of genes encoding ESBLs (e.g., *bla*_CTX − M−14_ and *bla*_CTX − M−15_) and carbapenemases (e.g., *bla*_KPC − 2_, *bla*_NDM − 1_, and *bla*_OXA − 48_). Colistin resistance associated with the *mcr-1* gene and substitutions in MgrB was reported in Saudi Arabia and the UAE. Some high-risk clones of *K. pneumoniae*, including the ST11, ST307, and ST340, were identified in specific geographical regions. These findings highlight substantial gaps, particularly in community-based, environmental, and molecular epidemiology studies, while limited molecular and genome-based analyses hinder comprehensive AMR surveillance.

Menezes et al. performed a prospective longitudinal study investigating the transfer of genes encoding broad-spectrum β-lactamases (i.e., ESBL, AmpC, and carbapenemase) of *Enterobacteriaceae* strains between companion animals and their cohabitant humans in Portugal and the United Kingdom (UK) during episodes of animal infection. Clinical specimens from dogs and cats, along with samples from their human cohabitants, were screened for antimicrobial-resistant strains, and whole-genome sequencing (WGS) was used to assess relatedness. ESBL/AmpC-producing strains were identified in companion animals and humans from Portugal and the UK, while carbapenemase-producing strains were detected in one dog from Portugal and another from the UK.

Furthermore, transmission of index clinical *E. coli* and *K. pneumoniae* strains producing ESBL to cohabitant humans occurred in three Portuguese households, with repeated isolation of these strains in fecal samples from both animals and their owners. Besides, longitudinal sharing of *E. coli* strains between companion animals and their owners was identified in two households from Portugal and two from the UK. Interestingly, an MDR *Enterobacter hormaechei* subsp. *hoffmannii* strain was also shared within another Portuguese household. These findings underscore the household as a key epidemiological unit in AMR dissemination and highlight the need for targeted antimicrobial surveillance to inform and evaluate public health interventions.

## Risk factors of hypervirulent and MDR strains

Convergence of hypervirulence and multidrug resistance poses a significant public health threat. In China, Zheng C. et al. conducted an active surveillance culture program from September 2020 to August 2021, screening 138 randomly selected pregnant women, with five undergoing sample collection at two different time points. A total of 41 carbapenem-resistant *Acinetobacter baumannii* (CRAB) strains were obtained and classified into four Cluster^RS^ groups and one orphan pattern. Cluster^RS^ 1, with eight complex types in pregnancy, was the dominant group globally, followed by Cluster^RS^ 13, identified as hypervirulent KL49 CRAB with a strong geographical association to Guangdong, China. The maternal CRAB carriage rate was 26.09%, with half of the strains representing novel complex types, including CT3071, marking the first identification of KL7 strains in Shenzhen, China. Strains assigned as KL49 and KL7 were most frequently found in the same participant, suggesting intraspecific competition as a potential mechanism for CRAB infections occurring without prior colonization in pregnancy. Independent risk factors for CRAB carriage included advanced maternal age, gestational diabetes mellitus, and Group B *Streptococcus* infection. The high carriage rate and enhanced virulence of CRAB in pregnancy underscore the urgent need for routine surveillance to prevent dissemination within this high-risk group, particularly for Cluster^RS^ 13 strains in Guangdong, China.

Liu N. et al. investigated the clonal replacement of clinical hypervirulent *K. pneumoniae* (hvKp) strains belonging to K2-ST881 and K54-ST29 lineages in a single patient experiencing multiple-site infections during two independent episodes. All strains were highly susceptible to multiple antimicrobial agents. Plasmid analysis revealed that both virulence plasmids, pEDhvKp-1 and pEDhvKp-3, belonged to the IncFIB-type and carried several virulence genes. Currently and early strains were closely related. By using a mouse infection model, K2-ST881 and K54-ST29 strains demonstrated high virulence, causing more than 90% mortality within 72 hours post-infection. These findings expand the known diversity of hvKp lineages, reveal genomic adaptations associated with clonal switching between distinct hvKp strains *in vivo*, and highlight the urgent need for improved surveillance, diagnostic tools, and therapeutic strategies to address the threat posed by high-risk hvKp strains.

In summary, the dissemination of MDR Gram-negative bacteria remains a critical public health challenge, driven by MGEs facilitating AMR across diverse environments. The presence of ESBL- and carbapenemase-producing strains at the human-animal-environmental interface highlights the urgent need for continuous surveillance and intervention strategies. The Guest Editors of this Research Topic hope these findings will inspire scientists to further explore and discuss the global spread and evolution of MDR Gram-negative bacterial pathogens.

